# Estrogen-Inducible LncRNA *BNAT1* Functions as a Modulator for Estrogen Receptor Signaling in Endocrine-Resistant Breast Cancer Cells

**DOI:** 10.3390/cells11223610

**Published:** 2022-11-15

**Authors:** Kuniko Horie, Kiyoshi Takagi, Toshihiko Takeiwa, Yuichi Mitobe, Hidetaka Kawabata, Takashi Suzuki, Kazuhiro Ikeda, Satoshi Inoue

**Affiliations:** 1 Division of Systems Medicine & Gene Therapy, Saitama Medical University, Saitama 350-1241, Japan; 2 Department of Pathology and Histotechnology, Tohoku University School of Medicine, Sendai 980-8575, Japan; 3 Department of Systems Aging Science and Medicine, Tokyo Metropolitan Institute of Gerontology, Tokyo 173-0015, Japan; 4 Department of Breast and Endocrine Surgery, Toranomon Hospital, Tokyo 105-8470, Japan; 5 Department of Anatomic Pathology, Tohoku University Graduate School of Medicine, Sendai 980-8575, Japan

**Keywords:** breast cancer, estrogen receptor, long noncoding RNA, endocrine therapy resistance, in situ hybridization, prognosis

## Abstract

Recent advances in RNA studies have revealed that functional long noncoding RNAs (lncRNAs) contribute to the biology of cancers. In breast cancer, estrogen receptor α (ERα) is an essential transcription factor that primarily promotes the growth of luminal-type cancer, although only a small number of lncRNAs are identified as direct ERα targets and modulators for ERα signaling. In this study, we performed RNA-sequencing for ER-positive breast cancer cells and identified a novel estrogen-inducible antisense RNA in the *COL18A1* promoter region, named *breast cancer natural antisense transcript 1* (*BNAT1*). In clinicopathological study, *BNAT1* may have clinical relevance as a potential diagnostic factor for prognoses of ER-positive breast cancer patients based on an in situ hybridization study for breast cancer specimens. siRNA-mediated *BNAT1* silencing significantly inhibited the in vitro and in vivo growth of tamoxifen-resistant ER-positive breast cancer cells. Notably, *BNAT1* silencing repressed cell cycle progression whereas it promoted apoptosis. Microarray analysis revealed that *BNAT1* silencing in estrogen-sensitive breast cancer cells repressed estrogen signaling. We showed that *BNAT1* knockdown decreased ERα expression and repressed ERα transactivation. RNA immunoprecipitation showed that *BNAT1* physically binds to ERα protein. In summary, *BNAT1* would play a critical role in the biology of ER-positive breast cancer by modulating ERα-dependent transcription regulation. We consider that *BNAT1* could be a potential molecular target for diagnostic and therapeutic options targeting luminal-type and endocrine-resistant breast cancer.

## 1. Introduction

Breast cancer is the most common malignancy for women, and its global incidence rate in 2020 was estimated to be approximately one fourth of female cancers worldwide [1]. Because the majority of breast cancers are primarily estrogen-dependent, understanding of estrogen-regulated gene network is the first step towards managing the disease [2]. Estrogen receptor α (ERα) is an essential transcription factor stimulated by estrogen [3], and the receptor also regulates the proliferation and progression of hormone-naïve breast cancer cells by modulating various ERα target genes [4]. Because the proliferation of estrogen-sensitive breast cancer cells can be managed by ERα antagonists, endocrine-related therapy using ERα antagonists such as tamoxifen has been performed for years for patients with ERα-positive breast cancer [4]. Long-term endocrine-related therapy, however, will often to the development of hormone-refractory tumors and patients will be eventually threatened by aggressive type of cancers [4]. Elucidation of estrogen signaling and identification of ERα target genes will facilitate in understanding the pathophysiology of breast cancer and to developing diagnostic and therapeutic options for the advanced disease.

Recent advance in RNA study reveals that various functional long noncoding RNAs (lncRNAs) contribute to the pathophysiology of cancers. We have previously identified that androgen-responsive lncRNA *CTBP1-AS* regulates the progression of prostate cancer cells by suppressing the expression of CTBP1 as well as various target genes including tumor suppressor genes [5]. Working together with RNA-binding proteins such as PSF, *CTBP1-AS* even promotes the progression of hormone-refractory prostate cancer cells that are maintained under a condition of long-term androgen deprivation. Considering that there are functional similarities between the androgen signaling in prostate cancer and the estrogen signaling in breast cancer, we next questioned if there is a critical lncRNA that is regulated by ERα and modulates ERα activities, leading to the progression of breast cancer cells.

In the regulatory system of estrogen signaling in breast cancer, several critical genomic regions have been identified as active enhancers, which function as key stations that orchestrate ER-dependent transcriptional regulation [6,7,8]. The lncRNAs associated with active enhancers have been defined as enhancer-associated lncRNAs or eRNAs [6,7], such as the lncRNAs in *CCND1* promoter region [9]. The identification of active enhancer-associated lncRNAs will provide critical information for precise understanding of the estrogen-regulated transcription in breast cancer pathophysiology, which leads to the development of new class of therapeutic options for the disease.

In the present study, we performed RNA-sequencing for ER-positive breast cancer cells and identified a novel critical estrogen-responsive lncRNA that modulates ERα-dependent transcriptional regulation. In the screening of estrogen-inducible lncRNAs transcribed from ERα-associated active promoters or enhancers, we discovered a novel estrogen-inducible lncRNA from the proximal promoter region of collagen XVIII gene *COL18A1*. We designated the lncRNA as *breast cancer natural antisense transcript 1* (*BNAT1*), which is transcribed from the vicinity of the transcriptional start site (TSS) region of *COL18A1*. *BNAT1* includes a functional ERα binding site in its RNA sequence and its expression is upregulated in tamoxifen-resistant MCF7 breast cancer cells compared with parental MCF7 cells. Our clinicopathophysiological study showed that intense signal of in situ hybridization for *BNAT1* is significantly associated with distant disease-free survival of patients with ER-positive breast cancer. The siRNA-mediated knockdown of *BNAT1* leads to the repression of ER-dependent transcription and the proliferation of ER-positive breast cancer cells. *BNAT1*-specific siRNAs could reduce the in vivo tumor formation derived from tamoxifen-resistant breast cancer cells. The modulation of *BNAT1* expression could alter ERα binding activity in the promoter regions of ER target genes. The present study defines that *BNAT1* is a pivotal estrogen-inducible lncRNA that could activate ERα signaling and promote breast cancer tumorigenesis. Our finding will provide new information for ER-dependent breast cancer progression and the development of alternative therapeutic options for endocrine therapy-resistant disease.

## 2. Materials and Methods

### 2.1. Cell Culture and Reagents

MCF7 human ER-positive breast cancer cells were obtained from the American Type Culture Collection (Manassas, VA, USA), cultured at 37 °C in a 5% CO_2_ humidified atmosphere using Dulbecco’s modified Eagle’s medium (DMEM) and RPMI, respectively, including 10% fetal bovine serum (FBS), 50 U/mL penicillin, and 50 μg/mL streptomycin. The MCF7 derivative cells resistant to 4-hydroxytamoxifen (OHT), or OHTR cells, were generated by being maintained in the medium including 1 μM OHT for >3 months [10]. Anti-ERα (H-184; Santa Cruz Biotechnology, Dallas, TX, USA) and anti-β-actin (A2228; Sigma-Aldrich, St. Louis, MO, USA) antibodies were utilized for immunoblotting.

### 2.2. qRT-PCR

Total RNA was extracted from cells using ISOGEN reagent (Nippon Gene, Toyama, Japan) and subjected to cDNA synthesis using Invitrogen SuperScript III reverse transcriptase (Thermo Fisher Scientific, Carlsbad, CA, USA) with random primers. Quantitative real-time PCR (qRT-PCR) was performed on the StepOnePlus system (Thermo Fisher Scientific) using a KAPA SYBR FAST quantitative PCR kit (KAPA Biosystems, Wilmington, MA, USA) as described previously [11]. RNA expression level was evaluated by the *ΔΔC_T_* method (*C_T_*, threshold cycle) and normalized to that of *RPLP0* in each sample. The primers used in this study are described in Table 1.

### 2.3. RNA-Sequencing

RNA-sequencing (RNA-seq) for MCF7 cells were conducted as described previously [11]. Briefly, cells were cultured in the hormone-depleted medium for 2 days and treated with either 100 nM of 17β-estradiol (E_2_), 100 nM E_2_ plus 1 μM OHT, or 0.1% ethanol as vehicle for 4 h. Libraries for RNA-seq were prepared using a SOLiD Total RNA-Seq Kit (Thermo Fisher Scientific) from poly(A)-selected RNAs. Single-end RNA-seq (50-bp read length) was performed on the Applied Biosystems SOLiD System 3.0 (Thermo Fisher Scientific) based on the standard protocol. Obtained fastq files were analyzed by Bowtie with the default option and the read tags were mapped to the human genome hg.19. Original RNA-seq data are deposited as a project id = 753 in a cloud-based data analysis platform, Maser (Management and Analysis System for Enormous Reads) [http://cell-innovation.nig.ac.jp/maser/, accessed on 29 August 2022], developed and managed by the National Institute of Genetics in Japan [12].

### 2.4. Clinical Tissue Samples and Patient Data

Clinical breast cancer tissue samples were obtained from a cohort of 115 Japanese female patients who had surgical treatment between 2006 and 2013 at Toranomon Hospital, Japan (age range from 31 to 76 years) [11]. Patients were not treated with chemotherapy or molecular target therapy before surgery. Standard adjuvant treatments were performed based on the Clinical Practice Guidelines of the National Comprehensive Cancer Network [13]. Staging of breast cancer was determined based on the TNM Classification of Malignant Tumours [14]. The clinical outcome was defined as distant disease-free survival using a time span from the date of surgery to the first distant recurrence or last follow-up. The mean follow-up duration was 83 months (ranging 8 to 118 months). This study was approved by the ethical committee at Toranomon Hospital (approval number 845) and Saitama Medical University International Medical Center institutional review board (approval number 13–148). All patients who participated in this study provided written informed consent. This study abided by the Declaration of Helsinki principles and was performed in accordance with the institutional guidelines and regulations for human experiments.

### 2.5. ISH

Digoxigenin (DIG)-labeled RNA probes were generated for in situ hybridization (ISH) using the DIG RNA Labeling Kit (Roche Diagnostics GmbH, Mannheim, Germany). RNA ISH was performed on formalin-fixed, paraffin-embedded (FFPE) breast cancer tissues as described previously [11]. Briefly, the slides were treated with proteinase K (Wako Pure Chemical Industries, Ltd., Osaka, Japan), refixed with 10% formalin, immersed in 0.2 N HCl for 10 min and hybridized with a *BNAT1* probe (25 ng per slide) in G-Hybo-L reagent (Genostaff, Tokyo, Japan) at 63 °C for 24 h [15]. For the detection of the ISH signal, the probe-hybridized slides were sequentially labeled with an anti-DIG mouse monoclonal antibody (Roche Diagnostics GmbH), a biotinylated anti-mouse IgG, and alkaline phosphatase-labeled streptavidin (Nichirei Bio, Inc., Tokyo, Japan). Finally, chromogenic signals were obtained using nitroblue tetrazolium–5-bromo-4-chloro-3-indolylphosphate solution (Roche, Switzerland) and counterstained by nuclear fast red. The intensity of the ISH signal was determined by a trained pathology technologist (K. Takagi) and pathologist (T. Suzuki).

### 2.6. siRNA Transfection

siRNAs targeting *BNAT1* and *ESR1* were designed using siDirect version 2.0 online software [http://sidirect2.rnai.jp/, accessed on 21 May 2016] and purchased from RNAi Inc. (Tokyo, Japan). A negative-control siRNA with no homology to known gene targets in mammalian cells, or siControl, was purchased from RNAi Inc. In siRNA transfection, cells were seeded at 300,000 cells per well in 6-well plates and simultaneously treated with a mixture of siRNA (final concentration: 10 nM) and Invitrogen Lipofectamine RNAiMAX (Thermo Fisher Scientific). Cells were harvested 48 h after transfection and subjected to qRT-PCR, cell cycle analysis, and apoptosis assay by annexin V and propidium iodide (PI) staining. The siRNAs used in these assays are described in Table 2.

### 2.7. DNA Assay

Cells were seeded at 1500 cells per well in 96-well plates with culture medium including 10%FBS. Cells were collected at 1, 3, and 5 day(s) after cell seeding and frozen. Cells were thawed and lysed with TNE buffer (10 mM Tris-HCl [pH 7.5], 2 mM NaCl, and 1 mM EDTA). Extracted DNA samples were stained with Hoechst 33258 pentahydrate (Thermo Fisher Scientific) at a final concentration of 5 µg/mL. The DNA content in each well was evaluated on the ARVO5 multimode plate reader system (PerkinElmer, Waltham, MA, USA) at 355 nm for 0.1 s.

### 2.8. Cell Cycle Analysis

siRNA-treated cells were harvested, fixed with 70% ethanol for >30 min, treated with RNase A, and then stained with 5 µg/mL PI. DNA contents were measured using the FACSCalibur platform (BD, Franklin Lakes, NJ, USA). Data were analyzed by CellQuest software (BD) to determine the percentage of cells in G1, S, and G2/M phases.

### 2.9. Annexin V and PI Staining

siRNA-treated cells were harvested and stained using a fluorescein isothiocyanate annexin V apoptosis detection kit (BD). Percentages of apoptotic cells were evaluated on the FACSCalibur platform.

### 2.10. In Vivo Tumor Formation and siRNA Treatment

All animal experiments were approved by the Animal Care and Use Committee of Saitama Medical University and carried out in accordance with the Guidelines and Regulations for the Care and Use of Experimental Animals of Saitama Medical University. Female nude mice were purchased from CREA Japan. OHTR cells were mixed with equal volumes of Matrigel matrix (Corning, Corning, NY, USA) and injected subcutaneously into the flanks of 8-week-old female nude mice. When the tumor volume reached 150 mm^3^, mice were divided in two groups randomly. siControl or s*iBNAT1* #B (5 µg each) was injected with Genlantis GeneSilencer siRNA Transfection Reagents (Genlantis, San Diego, CA, USA) into the tumors twice a week. Three dimensions of tumors were measured once a week, and tumor volumes were estimated with the following formula: 0.5 × 1st diameter × 2nd diameter × 3rd diameter.

### 2.11. Microarray and Pathway Analysis

Microarray analysis was performed using human Clariom D array (Thermo Fisher Scientific) based on the manufacturer’s instructions. Data were analyzed using Affymetrix Expression Console software build 1.4.1.46 (Thermo Fisher Scientific). Pathway analyses were performed using Gene Set Enrichment Analysis 3.0 (GSEA) developed by the GSEA/MSigDB Team consisting of members from the Broad Institute (Cambridge, MA, USA) and University of California, San Diego, (CA, USA) [https://www.gsea-msigdb.org/gsea/index.jsp, accessed on 20 November 2017] and the DAVID Functional Annotation Clustering Tool 2021 developed by Laboratory of Human Retrovirology and Immunoinformatics (Frederick National Laboratory for Cancer Research, Frederick, MD, USA) [http://david.abcc.ncifcrf.gov/summary.jsp, accessed on 14 June 2022].

### 2.12. Immunoblot Analysis

Cells were suspended in Laemmli sample buffer (125 mM Tris-HCl [pH 6.8], 20% glycerol, 4% sodium dodecyl sulfate [SDS], 5% 2-mercaptoethanol, and bromophenol blue) and boiled for 10 min at 100 °C. Immunoblot analysis was performed as described previously [11].

### 2.13. RNA Immunoprecipitation (RIP) Assay

RIP assay was performed as described previously [16,17]. Briefly, cells were crosslinked with formaldehyde at a final concentration of 0.3% for 5 min and quenched by glycine at a final concentration of 36 mM for 5 min at room temperature. After washing with PBS, cells were scraped and resuspended in hypotonic buffer (10 mM HEPES pH 7.9, 10 mM KCl, 0.1 mM EDTA, 0.1 mM EGTA, 0.6% NP-40, 1 mM DTT, 1 mM PMSF, and 3 μg/mL of aprotinin). Nuclear pellets were collected by the centrifugation at 4 °C, 15,000 rpm for 15 min, and then resuspended in RIP buffer (150 mM KCl, 25 mM Tris–HCl pH 7.4, 5 mM EDTA, and 0.5% NP-40) and lysed by 10 strokes with a 26G needle. Nuclear extracts were centrifuged at 15,000 rpm for 15 min at 4 °C, and the supernatants were mixed with an anti-ERα antibody (H-184; Santa Cruz Biotechnology) or a control rabbit IgG and rotated overnight at 4 °C. ERα-RNA complexes were precipitated using Protein G Sepharose 4 Fast Flow (Cytiva, Marlborough, MA, USA), and bead-conjugated RNAs were eluted using Sepasol-RNA I Super G (Nacalai Tesque, Kyoto, Japan).

### 2.14. Luciferase Assay

Cells (50,000 cells/well in a 24-well plate) cultured in the hormone-depleted medium for 1 day were transfected with the following luciferase vectors together with Lipofectamine 2000 reagent (Thermo Fisher Scientific): *Firefly* luciferase vectors (psiCHECK2 vector [Promega Corporation, Fitchburg, WI, USA] or ERE-luciferase vectors, 300 ng each) and *Renilla*-expressing vector (10 ng). Cells were treated with either 10 nM of E_2_ for 24 h and harvested 48 h after transfection and luciferase activities were evaluated using the Dual-Luciferase reporter assay system (Promega Corporation) on the TriStar2 S LB942 system (Berthold Technologies GmbH KG & CO, Bad Wildbad, Germany).

### 2.15. Statistical Analysis

Statistical analysis was performed using Excel Statistics 2010 (add-in software for Microsoft Excel) (SSRI Co., Ltd., Tokyo, Japan). The correlation between *BNAT1* status and clinicopathological factors was evaluated by the Student’s *t*-test or Pearson’s chi-squared (χ^2^) test. Distant disease-free survival curve was generated by the Kaplan–Meier method and the *p*-value was evaluated by the log-rank test. Candidates of affecting factors on distant disease-free survival were evaluated by univariate Cox proportional regression analysis. Only significant factors in the univariate analysis were used for multivariate Cox proportional regression analysis, and significance was determined at *p*-value < 0.05 following Bonferroni correction. Statistical significance in in vitro and in vivo experiments was determined by the Student’s *t*-test or analysis of variance, respectively. All the data in the text and figures are presented as means ± standard error of the mean (SEM).

## 3. Results

### 3.1. Identification of the Estrogen-Inducible LncRNA BNAT1

**Figure 1:**
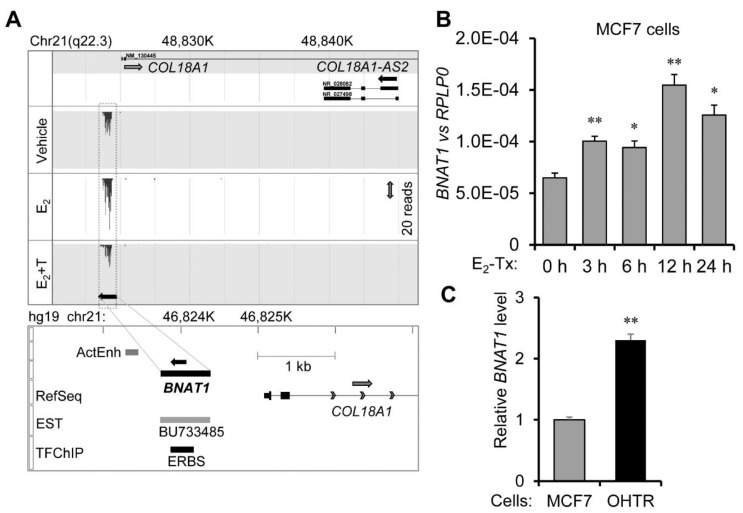
Estrogen-inducible lncRNA *breast cancer natural antisense transcript 1* (*BNAT1*) is transcribed from the promoter region of *COL18A1* and expressed in estrogen receptor (ER)-positive breast cancer cells. (**A**) Mapping of RNA-seq read tags from MCF7 cells to the antisense strand adjacent to the *COL18A1* locus in chromosome 21q22.3. *BNAT1* expression was enhanced in MCF7 cells with 17β-estradiol (E_2_, 100 nM) treatment for 4 h in comparison to vehicle or E_2_ (100 nM) + T (OH-tamoxifen, 1 µM) treatment for 4 h. ActEnh, a cluster region including at least three E_2_-upregulated active enhancers (previously defined in MCF7 cells by refs. [6,7,8], *n* = 1248) with a distance between two adjacent enhancers ≤ 25 kb. Detailed information is described in Table S1. ERBS, a functional ER-binding site (ERBS) defined by the ENCODE dataset of ChIP-seq in E_2_-treated endometrial cancer ECC cells (GEO accession number: GSM803422). (**B)** E_2_ (100 nM)-dependent upregulation of *BNAT1* in MCF7 cells. (**C**) *BNAT1* was overexpressed in OH-tamoxifen-resistant MCF7 (OHTR) cells compared with parental MCF7 cells. *, *p* < 0.05; **, *p* < 0.01.

While some *cis*-acting enhancer-associated lncRNAs were previously identified in ER-positive breast cancer cells (e.g., lncRNAs in *CCND1* promoter [9]), little is known in terms of lncRNAs that are activated by ERα and that directly activate ERα signaling. To dissect novel estrogen-dependent lncRNAs in breast cancer cells, we performed RNA-sequencing (RNA-seq) in ER-positive MCF7 cells treated with 17β-estradiol (E_2_, 100 nM), E_2_ (100 nM) with OH-tamoxifen (OHT, 1 µM), or a control vehicle for 4 h. Cells were maintained in hormone-deprived culture medium for 2 days prior to ligand treatment. Previous studies by Prof. Lee Kraus’ and Prof. Rosenfeld’s groups defined estrogen-induced “active enhancers” in MCF7 cells [6,7,8]. Among 1248 E_2_-upregulated active enhancers, we focused on 66 clusters including at least 3 active enhancers per a region, with a distance between 2 adjacent enhancers ≤ 25 kb (Table S1). We expected to identify functional estrogen-inducible lncRNAs from these active enhancer cluster regions, because the previous screening of target genes for ERα or the androgen receptor was often performed with a range of 20–100 kb from the receptor binding sites [18,19]. In the screening of significant E_2_-inducible lncRNAs in the vicinity of active enhancer cluster regions based on RNA-seq data, we discovered a substantial E_2_-upregulated transcript derived from a genomic region including a functional ER-binding site (ERBS), which is located in the proximal upstream region of the *COL18A1* gene in the antisense direction based on the ENCODE dataset of chromatin immunoprecipitation sequencing (ChIP-seq) in E_2_-treated endometrial cancer ECC cells retrieved in the UCSC Genome Browser (hg19, chr21:46823868–46824171, GEO accession number: GSM 803422) (Figure 1A). Although no annotated transcript was identified in the RefSeq database, several EST transcripts have been identified in the vicinity of the region (Figure 1A).

We designated the E_2_-inducible lncRNA identified in this region as “*breast cancer natural antisense transcript 1*”, or *BNAT1*. The RNA-seq read tags showed that the sequence of *BNAT1* corresponds to the reverse complement of the expressed sequence tag (EST) BU733485 identified in either E_2_-treated or control MCF7 cells (Figure S1A). The GC content of the identified sequence for *BNAT1* is 62%. The transcript includes a particular GC-rich sequence in its 5′-terminal region, whereas it includes an AT-rich sequence in its 3′-terminal region (the GC content for the first and last 50-nt is 76% and 24%, respectively). In silico analysis for RNA secondary structure predicts a stem and loop secondary structure for *BNAT1* (Figure S1B), based on minimum free energy prediction in the RNAfold web server operated by the University of Vienna [http://rna.tbi.univie.ac.at//cgi-bin/RNAWebSuite/RNAfold.cgi, accessed on 29 August 2022]. Intriguingly, the RNA contains a canonical ER binding motif in its sequence (highlighted in yellow in Figure S1A) as analyzed by the JASPAR database for curated and non-redundant transcription factor (TF) binding profiles [https://jaspar.genereg.net/, accessed on 29 August 2022]. A recent study by Prof. Ruggero and colleagues further revealed that ERα is a non-canonical RNA-binding protein (RBP), which preferentially binds to a 5-nt motif sequence U[A/G]AU [C/U] as determined by ERα ultraviolet radiation cross-linking and immunoprecipitation (CLIP) [20]. We found that the *BNAT1* sequence included two ERα-bound consensus motifs as highlighted in green in Figure S1A.

The expression of *BNAT1* was rapidly induced by E_2_ treatment in ER-positive MCF7 cells (Figure 1B). We have previously generated OHT-resistant MCF7 (OHTR) cells under ≥3-month culture of MCF7 cells in OHT-containing medium [10]. Notably, *BNAT1* expression was upregulated in OHTR cells compared with the parental MCF7 cells (Figure 1C), suggesting that the transcript plays a pathophysiological role in the progression of hormone-dependent breast cancers.

### 3.2. Correlation between BNAT1 Expression Levels and Clinicopathologic Characters in ER-Positive Breast Cancers

**Figure 2:**
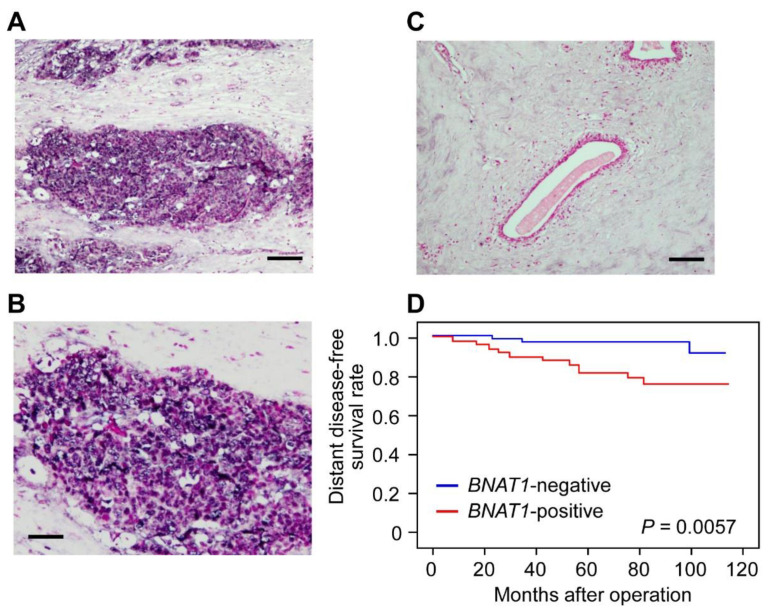
*BNAT1* expression in breast cancer tissues were positivity correlated with poor prognoses of the patients. (**A**–**C**) Representative results of in situ hybridization (ISH) analysis for *BNAT1* in malignant (**A** for low magnitude, **B** for high magnitude) and benign (**C**) mammary tissues. Scale bars, 100 μm for **A**, **C**, 50 μm for **B**. (**D**) Kaplan–Meier plot analysis showing the relationship between *BNAT1* ISH signals in cancer tissues and distant disease-free survival of breast cancer patients (blue, ISH negative, *n* = 66; red, ISH positive, *n* = 49).

We next investigated whether *BNAT1* is expressed in ER-positive clinical breast cancers and whether it is associated with the prognoses of patients. We examined *BNAT1* expression in 115 clinical breast cancer specimens that were excised from patients as primary breast cancer tissues and previously defined as ER-positive by an immunohistochemical study [11]. As analyzed by in situ hybridization (ISH) for *BNAT1*, we found that intense ISH signals were often detected in the nucleus and cytoplasm of solid tumor lesions and were determined as ISH positive (Figure 2A,B), whereas weak or negative signals were shown in non-cancerous regions around benign mammary ducts (Figure 2C). Based on the abovementioned criteria for ISH positivity, 49 and 66 of the 115 patients (43%) had tumors with a positive and negative ISH signal of *BNAT1*, respectively (Table 3). *BNAT1* positivity was significantly associated with pathological T factor (pT; *p* = 0.017) (Table 3). We next assessed the relationship between *BNAT1* positivity and the clinical prognosis of breast cancer patients. *BNAT1* positivity was significantly correlated with poorer distant disease-free survival based on analysis by Kaplan–Meyer plot analysis (Figure 2D). The correlation between *BNAT1* positivity and overall survival was not determined because no patients with a *BNAT1*-negative tumor died during the observation period.

Univariate analysis using the Cox proportional hazard model showed that *BNAT1* status could be a significant prognostic factor, along with known prognostic factors pN (pN0–1 versus pN2–3) and pT (pT1 versus pT2–4) status, based on pathological TNM stage for distant disease-free survival (Table 4). Multivariate analysis of Cox regression model was performed among pN, pT, and *BNAT1* status as these factors were statistically significant in univariate analysis. Multivariate analysis showed that *BNAT1* ISH positivity and pN status were independent prognostic factors for distant disease-free survival (Table 4).

### 3.3. BNAT1 Silencing Represses Cell Proliferation whereas It Promotes Apoptosis in Estrogen-Sensitive and Endocrine-Resistant Breast Cancer Cells

**Figure 3:**
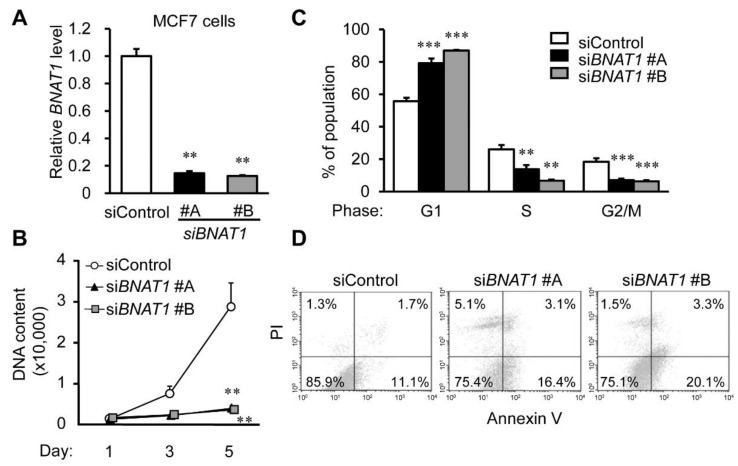
*BNAT1* silencing represses the viability and cell cycle progression, whereas it promotes apoptosis of hormone-refractory breast cancer cells. (**A**) Knockdown efficiency of *BNAT1* siRNAs (si*BNAT1*s) #A and #B compared with control siRNA (siControl) in MCF7 cells analyzed by qRT-PCR (*n* = 3). (**B**) The viability of MCF7 cells treated with indicated siRNAs, analyzed by DNA assay. Values are presented as means ± SEM versus levels for siControl in each cell type (*n* = 5). (**C**) Cell cycle distribution of siRNA-treated MCF7 cells stained with propidium iodide (PI) analyzed by flow cytometry. Percentages of cell populations in G1, S, and G2/M phases are shown (*n* = 3). (**D**) Percentages of annexin V-positive populations in MCF7 cells treated with the indicated siRNAs, analyzed by flow cytometry (*n* = 3). **, *p* < 0.01; ***, *p* < 0.001.

To understand the function of *BNAT1* in ER-positive breast cancer cells, we transfected siRNAs targeting *BNAT1* to estrogen-sensitive MCF7 and endocrine-resistant model OHTR cells. The siRNAs targeting *BNAT1* si*BNAT1* #A and #B could substantially downregulate *BNAT1* expression in MCF7 and OHTR cells (Figure 3A and Figure S2A). *BNAT1* silencing significantly suppressed the proliferation of these cells (Figure 3B and Figure S2B) and decreased the percentages of S-phase cells (Figure 3C and Figure S2C). Moreover, siRNA-mediated *BNAT1* knockdown increased the percentages of apoptosis-related annexin V-positive fractions (Figure 3D and Figure S2D). We further generated OHTR-derived xenograft models from female nude mice and evaluated the therapeutic effects of siRNAs targeting *BNAT1* (Figure S2E). siRNAs were intratumorally injected twice weekly into nude mice inoculated with OHTR cells from the time point when the xenografted tumors reached a volume of 150 mm^3^. si*BNAT1* injection significantly suppressed the growth of OHTR-derived xenograft tumors (Figure S2E). The administration of siRNAs specific to *BNAT1* could reduce the in vivo tumor growth of OHTR-derived xenografts, thus exhibiting their therapeutic effects on endocrine-resistant breast cancer.

### 3.4. BNAT1 Silencing Represses Estrogen Signaling and BNAT1 Preferentially Binds to ERα

**Figure 4:**
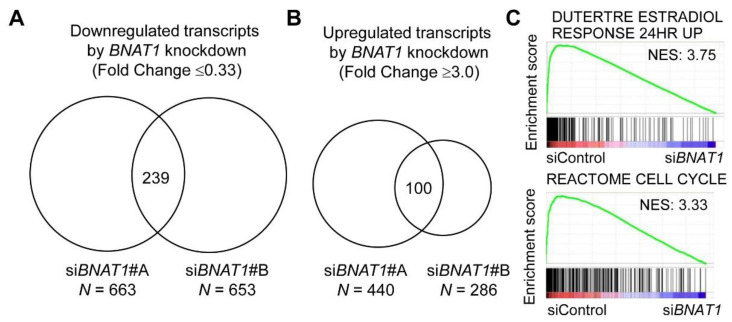
Differentially expressed transcripts in MCF7 cells treated with *BNAT1*-specific siRNAs versus control siRNA. (**A**) Downregulated transcripts by si*BNAT1* #A or #B versus siControl with a fold change ≤ 0.33. (**B**) Upregulated transcripts by si*BNAT1* #A or #B versus siControl with a fold change ≥ 3.0. (**C**) Gene Set Enrichment Analysis (GSEA) plots for dominant pathways in MCF7 cells treated with si*BNAT1*s #A and #B versus siControl, including the signaling for estradiol response and endocrine therapy resistance.

To determine the effects of *BNAT1* in ER-positive breast cancer biology, we performed microarray analysis in MCF7 cells transfected with either si*BNAT1* #A, si*BNAT1* #B, or control siRNA (siControl). In terms of downregulated transcripts in MCF7 cells by *BNAT1* silencing (fold change ≤ 0.33 versus siControl treatment), we selected 663 and 653 transcripts from RNAs obtained from cells transfected with si*BNAT1* #A and #B, respectively, compared with RNA from siControl-treated cells (Figure 4A). Among the 239 transcripts commonly downregulated by si*BNAT1* #A and #B, Gene Ontology (GO) terms for biological processes related to cell proliferation, including cell division, mitotic sister chromatid segregation, and the cell cycle, were enriched based on the functional enrichment analysis by DAVID Functional Annotation Bioinformatics (Table 5). In terms of upregulated transcripts by *BNAT1* silencing (fold change ≥ 3.0 versus siControl treatment), 440 and 286 transcripts from cells treated with si*BNAT1* #A and #B were selected, respectively, compared with cells treated with siControl (Figure 4B). Among the 100 transcripts commonly upregulated by si*BNAT1* #A and #B, we showed that GO terms related to cell-cell adhesion and wound healing were enriched as analyzed by DAVID (Table 6).

Moreover, pathway analysis based on Gene Set Enrichment Analysis among 34,246 genes showed that *BNAT1* silencing was remarkably associated with the repression of estrogen signaling and cell cycle-related pathways (Figure 4C).

**Figure 5:**
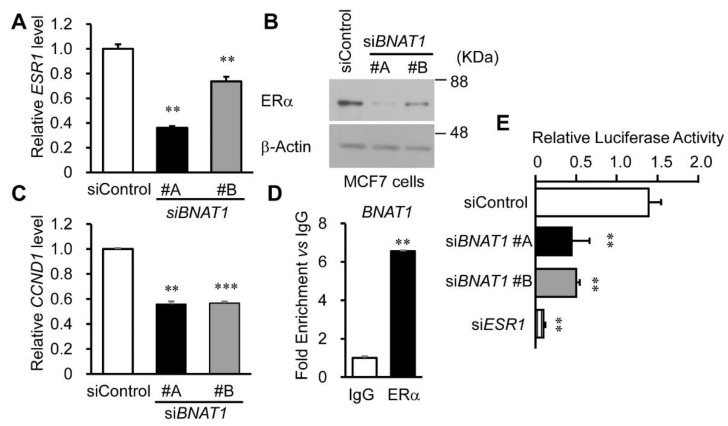
*BNAT1* silencing attenuates ERα expression and signaling in MCF7 cells (**A**) Downregulation of *ESR1* expression in MCF7 cells treated with si*BNAT1*s. Relative RNA levels are presented as mean fold changes ± SEM versus levels for siControl (*n* = 3). (**B**) Immunoblotting of ERα protein in MCF7 cells treated with si*BNAT1*s. (**C**) Effect of *BNAT1* silencing on the *CCND1* level in MCF7 cells. Relative RNA levels are presented as mean fold changes ± SEM versus levels for siControl (*n* = 3). (**D**) ERα protein physically interacts with *BNAT1* in MCF7 cells. RNA immunoprecipitation assay was performed using an anti-ERα antibody or a normal rabbit IgG. *BNAT1* mRNA level was analyzed by qRT-PCR. The fold enrichment relative to IgG was shown as means ± SEM (*n* = 3). (**E**) *BNAT1* silencing suppresses E_2_ (10 nM, 24 h)-dependent estrogen-responsive element (ERE)-based luciferase activity in MCF7 cells. Relative luciferase activity, determined by the normalization of *Firefly* luciferase value to *Renilla* luciferase value, are presented as mean fold changes ± SEM versus levels for siControl (*n* = 3). si*ESR1*, *ESR1*-specific siRNA. **, *p* < 0.01; ***, *p* < 0.001.

We next examined whether *BNAT1* silencing influences ERα expression. We showed that si*BNAT1*s substantially downregulated the *ESR1* mRNA level (Figure 5A and Figure S3A) as well as the ERα protein level (Figure 5B and Figure S3B) in MCF7 and OHTR cells. Among the genes involved in the estrogen signaling pathway, we showed that *CCND1* expression was significantly downregulated in MCF7 and OHTR cells transfected with si*BNAT1* #A or #B compared with those transfected with siControl (Figure 5C and Figure S3C). We questioned whether *BNAT1* directly binds to the ERα protein. We showed that *BNAT1* expression was remarkably enriched in RNA samples obtained from MCF7 cells immunoprecipitated by an anti-ERα antibody compared with a nonimmune IgG (Figure 5D). In a luciferase assay based on an estrogen response element reporter gene, the E_2_-dependent ERα transactivation activity in MCF7 cells transfected with si*BNAT1* #A or #B was significantly decreased compared with that in cells transfected with siControl (Figure 5E). Taken together, *BNAT1* can physically interact with ERα and the silencing of *BNAT1* downregulates ERα expression, leading to the repression of estrogen signaling in ER-positive breast cancer cells.

## 4. Discussion

In the screening of estrogen-inducible lncRNAs in ER-positive breast cancer cells, we identified a novel RNA *BNAT1* in the proximal promoter region of *COL18A1*. In situ hybridization analysis showed that *BNAT1* positivity in ER-positive breast cancer tissues was significantly correlated with poorer patient prognosis in comparison to *BNAT1* negativity. *BNAT1* positivity was an independent prognostic factor for ER-positive breast cancer patients based on multivariate analysis. The siRNA-mediated *BNAT1* silencing in ER-positive MCF7 cells as well as in tamoxifen-resistant OHTR cells exhibited the inhibition of cell proliferation and cell cycle progression, and the promotion of apoptosis. In the OHTR-derived xenograft model, the intratumoral injection of *BNAT1*-specific siRNA significantly repressed in vivo tumor growth. Microarray analysis in MCF7 cells showed that *BNAT1* silencing repressed estrogen signaling and endocrine therapy resistance based on GSEA pathway analysis. We showed that *BNAT1*-specific siRNAs significantly downregulated the expression of *CCND1*, a prototypic ERα target gene that drives cell cycle progression [21]. Notably, CCND1 is often abundantly expressed or amplified in ER-positive breast cancer [22]. Because high expression of CCND1 was reported to be associated with poor prognosis in a subset of ER-positive breast cancer patients [23], we assume that *BNAT1* silencing may be applied to alternative therapeutic options for endocrine-resistant breast cancers through the inhibition of the CCND1-dependent cell proliferation pathway. Moreover, RNA immunoprecipitation for ERα in MCF7 cells showed that *BNAT1* is an RNA that physically binds to ERα protein, and *BNAT1* silencing suppressed estrogen-dependent ERα transactivation as analyzed by luciferase assay using a reporter gene including an estrogen response element. Overall, *BNAT1* is a functional lncRNA that regulates ERα transcriptional activity and contributes to estrogen-dependent cell proliferation and tumor formation in ER-positive breast cancer.

In ER-positive breast cancer cells, endocrine therapy resistance often occurs through the enhancement of estrogen signaling, which can be brought on by factors such as ERα overexpression/amplification, acquired gain-of-function mutations of ERα, and altered interactions between ERα and coregulators [24]. We assume that the consensus sequence for the ERα-binding motif in *BNAT1* may facilitate ERα transactivation at the promoters/enhancers of ERα target genes including *ESR1* itself through the amplification of ERα protein recruitment to ERα binding sites in open chromatin regions, leading to the formation of transcriptional active complexes including ERα-related coactivators.

We previously showed that *TMPO-AS1* is a proliferative-related lncRNA in breast cancer and can be a prognostic factor for patients with breast cancer [11,25]. In ER-positive breast cancer cells, *TMPO-AS1* is considered to prolong the stability of *ESR1* mRNA by physically binding to *ESR1* mRNA through its complementary sequence [11]. *TMPO-AS1* was also overexpressed in tamoxifen-resistant OHTR cells compared with parental MCF7 cells and the silencing of *TMPO-AS1* could repress the in vivo tumor growth derived from OHTR cells [11]. In contrast to *TMPO-AS1*, our RIP analysis showed that *BNAT1* may physically interact with the ERα protein, putatively providing a scaffold for the transcriptional activation of ERα complexes. ERα has been recently defined as a functional RBP, which modulates post-transcriptional regulation [20]. For example, ERα RNA-binding is important for the alternative splicing of transcription factor X-box binding protein (XBP1), and the short-spliced isoform of XBP1 (*XBP1s*) was shown to be overexpressed in tamoxifen-resistant MCF7 cells [20]. In contrast to XBP1, ERα RNA-binding also regulates the translation of the *eIF4G2* and *MCL1* mRNAs, whereas it does not initially alter the expression of these mRNAs [20]. Notably, we found that the expression of *XBP1s* (NM_001079539) was remarkably downregulated, whereas the expression of *eIF4G2* and *MCL1* mRNAs was stable or rather upregulated in MCF7 cells treated by si*BNAT1* versus siControl in our microarray analysis. The transcriptomic study may indicate that *BNAT1* may contribute to ERα RNA binding and ERα-mediated RNA metabolism in ER-positive breast cancer.

It has been recently shown that a cluster of *ESR1*-associated lncRNAs, or *ESR1* locus enhancing and activating non-coding RNAs (*Eleanors*), were transcribed from a large chromatin domain containing the *ESR1* locus and the expression of *Eleanors* was particularly upregulated in long-term estrogen deprivation (LTED) MCF7-derived cells [26]. Such a cluster of lncRNAs may also contribute to the formation of topologically associating domains (TADs) [27]. Moreover, the *ESR1*-*Eleanor* TAD on 6q25 can interact with another transcriptionally active TAD that transcribes *FOXO3* on 6q21, and the expression of *Eleanor* contributes to the long-range chromatin interaction between *ESR1* and *FOXO3* in LTED cells [27]. While *BNAT1* is located on chromosome 21, questions may arise as to whether other *ESR1*-modulating factors on different chromosomes including *TMPO-AS1* and *Eleanors* contribute to the expression of *BNAT1* in breast cancer cells. Further studies may define the role of *BNAT1* in differential chromosomal interactions in ER-positive endocrine-resistant breast cancer cells.

## 5. Conclusions

This study showed the pathophysiological role of a novel estrogen-inducible lncRNA *BNAT1* in ER-positive and endocrine-resistant breast cancer cells. *BNAT1* ISH positivity was significantly correlated with the prognoses of patients with ER-positive breast cancers. *BNAT1* silencing inhibited in vitro and in vivo growth of endocrine-resistant breast cancer cells by repressing ERα expression and signaling. Our findings indicate that *BNAT1* could be a promising diagnostic and therapeutic factor for ER-positive and endocrine-resistant breast cancers.

## Figures and Tables

**Table 1:** Primers used in this study.

Gene	Forward Primer	Reverse Primer
*BNAT1*	GCCAGGGCTCATCTCCTATG	GGAGATCAGGGAGACTGGAGACT
*RPLP0*	CCACGCTGCTGAACATGCT	GATGCTGCCATTGTCGAACA
*ESR1*	AGACGGACCAAAGCCACTTG	CCCCGTGATGTAATACTTTTG
*CCND1*	TGCCCTCTGTGCCACAGAT	CCGCTGCCACCATGGA

**Table 2:** siRNAs used in this study.

siRNA	Sense	Antisense
*siBNAT1 #A*	GCACUGUGAGGAAGGAAAUTT	AUUUCCUUCCUCACAGUGCTT
*siBNAT1 #B*	GGACCAAACAGCGUAGUCUCC	AGACUACGCUGUUUGGUCCAG
*siESR1*	GCCUAGCUUGCCGUAAUGAUU	UCAUUACGGCAAGCUAGGCAA

**Table 3:** Association between *BNAT1* status and clinicopathological factors in 115 ER-positive breast carcinomas.

Parameter	*BNAT1* Status		*p*-Value
	+ (*n* = 49)	– (*n* = 66)	
**Age ^1^ (years)**	52.7 ± 1.7	53.3 ± 1.4	0.78
Stage			
I	19	40	
II	26	24	
III	4	2	0.054
Pathological T factor (pT)			
pT1	24	18	
pT2–4	25	48	**0.017**
Pathological N factor (pN)			
pN0–1	34	54	
pN2–3	15	12	0.12
Histological grade			
1–2	43	57	
3	6	9	0.83
HER2 status			
Positive	7	6	
Negative	42	60	0.38

^1^ Data are presented as means ± SEM. All other values represent the number of cases. *p*-value < 0.05 and 0.05 ≤ *p*-value < 0.10 were significant (in bold) and borderline significant (in italics), respectively.

**Table 4:** Univariate and multivariate analyses of distant disease-free survival in 115 ER-positive breast cancer patients.

Variable	Univariate *p*-Value	Multivariate *p*-Value ^2^	Relative Risk (95% CI)
pN	**0.0019 ^1^**	**0.015**	3.53 (1.28–9.72)
(pN0–1 vs. pN2–3)			
pT	**0.0071 ^1^**	*0.072*	2.73 (0.92–8.19)
(pT1 vs. pT2–4)			
BNAT1 status	**0.011 ^1^**	**0.046**	3.23 (1.02–10.22)
(negative vs. positive)			
HER2 status	0.45		
(negative vs. positive)			
Histological grade	0.50		
(1,2 vs. 3)			
Age	0.70		
(<50 vs. 50)			

The details for the collection of ER-positive breast cancer tissue samples and the clinical data for 115 patients were previously described [11]. Statistical analysis was evaluated by a proportional hazard model (Cox). ^1^ Significant (*p* < 0.05) univariate values were examined in the multivariate analyses in this study. ^2^ *p*-value < 0.05 and 0.05 ≤ *p*-value < 0.10 for multivariate values were considered significant and borderline significant and were listed in bold and italics, respectively. 95% CI, 95% confidence interval.

**Table 5:** Top 5 Gene Ontology (GO) terms among 239 commonly downregulated transcripts by si*BNAT1* #A and #B.

GO ID	Term	*p*-Value
GO:0051301	cell division	2.1E-44
GO:0000070	mitotic sister chromatid segregation	1.5E-19
GO:0007059	chromosome segregation	2.1E-18
GO:0006334	nucleosome assembly	2.5E-17
GO:0007049	cell cycle	1.0E-15

**Table 6:** Top 5 Gene Ontology (GO) terms among 100 commonly upregulated transcripts by si*BNAT1* #A and #B.

GO ID.	Term	*p*-Value
GO:0098742	cell-cell adhesion via plasma–membrane adhesion molecules	5.50E-04
GO:0042060	wound healing	8.50E-04
GO:0007275	multicellular organism development	2.00E-03
GO:0034332	adherens junction organization	5.50E-03
GO:0007043	cell-cell junction assembly	1.10E-02

## Data Availability

Original microarray data are available from the Gene Expression Omnibus (GEO) database with the accession number GSE212683. Original RNA-seq data are deposited as a project id = 753 in a cloud-based data analysis platform, Maser (Management and Analysis System for Enormous Reads) [http://cell-innovation.nig.ac.jp/maser/, accessed on 21 January 2016], developed and managed by the National Institute of Genetics in Japan, and are available from the platform manager on reasonable request [cip-contact@cello.lab.nig.ac.jp].

## Supplementary Materials

The following supporting information can be downloaded at: https://www.mdpi.com/article/10.3390/cells11223610/s1, Figure S1: The sequence of lncRNA *BNAT1* and its putative secondary structure; Figure S2: *BNAT1* silencing represses the viability and cell cycle progression, whereas it promotes apoptosis of hormone-refractory breast cancer OHTR cells; Figure S3: Effects of *BNAT1* silencing on the expression of ERα and its target gene *CCND1* in hormone-refractive OHTR cells; Table S1: Estrogen-inducible active enhancer clusters identified from a subgroup of ERα/ acetylated histone H3K27 co-bound enhancers in MCF7 cells previously published in the literature [6,7,8].
