# Supplementary material for: Estrogen-Inducible LncRNA BNAT1 Functions as a Modulator for Estrogen Receptor Signaling in Endocrine-Resistant Breast Cancer Cells
Source: Cells. 2022 Nov 15;11(22):3610. doi: 10.3390/cells11223610 (PMC9688125; doi:10.3390/cells11223610)
Supplement: Supplementary file 1 [file cells-11-03610-s001.zip › cells-2007077-supplementary.pdf]

Supplementary Materials

A

```
>hg19_dna range=chr21:46823721-46824412 strand=-
GGGCCAGCATCCTGAGCCCCACCTGCAGGCCCTGGGCGCTTTCCTCCTGG
CAGCTGTGCCCCCACCCGACCGGACCGCTCCCTGCAACCAGAGAGCCCTC
ACACCAGCAACACCCCCCAGCCTGCTCCCTCCTCGTGTGCGCGAAACTCA
TGGCACTGGCTTGACGGCTTTCTCTGAAACCCTCTGATTCCGCCTGGCCC
ATCCACACTGTCCCTTCCCGGCCAGGGCTCATCTCCTATGCCTGGACCAA
ACAGCGTAGTCTCCAGTCTCCCTGATCTCCCCACACGTGGCCCCACCCAT
CCTCTGAAGCAGCCCCAGGAGGCAGCAGTGACTTCACACTTCTAGTGCCC
GGCCCTCCCGGCCCTCCTCTCTGGGCTTGCTTGCCAGGGTCCTCCCAACC
TGACTGCTGCCTCCCACCTCCTCTCCGCCCCTGGAGCTCTCCAGCCGCAG
CCGCGGGGCCTGCTTGGGATTCTCCTCTGCTGGCATCATCCCTGCCTGCC
TCAGCCTTCCCAGGCCCAATCCTGCCCGATCTCACCTCTTCCCGCCCAGC
CACCTGGTCGGGTCCCGACCAGCATCTGCCTTCAAACCTGGAGTCCTTCC
CTGAGTGCTTGCGGTGCACTGTGAGGAAGGAAATCAATGTAAAGGATTAT
GACCCACATTTTTTAAAAATAAAAAGATTAAGGAGAACAGCA
```

B

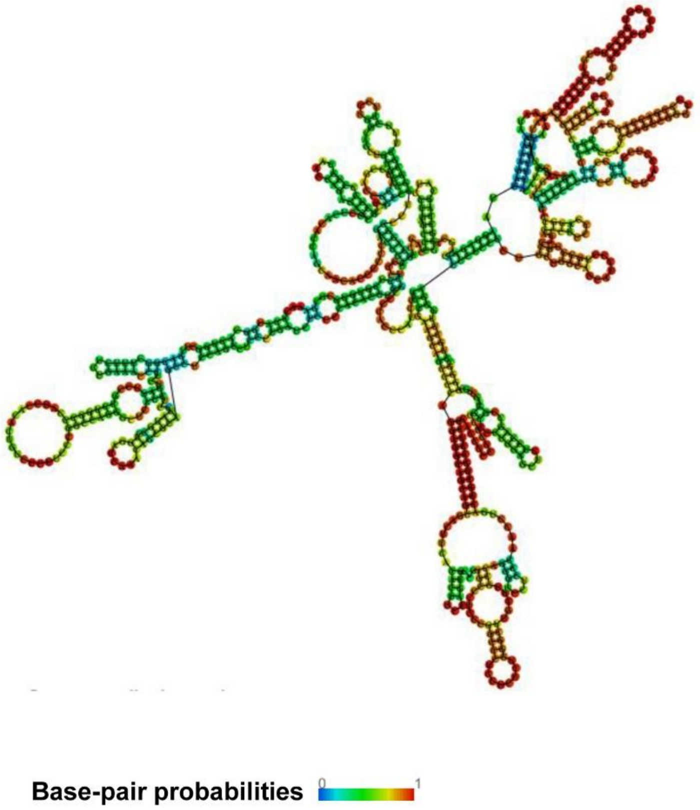

Figure S1. The sequence of lncRNA *BNAT1* and its putative secondary structure. (**A**) Sequence of *BNAT1* determined by RNA-seq for MCF7 cells. Highlighted bases in yellow and green correspond to a canonical ER binding motif as a transcription factor as defined by JASPAR database [https://jaspar.genereg.net/] and two consensus motifs for ERα as an RNA-binding protein as defined by Prof. Ruggero and colleagues [ref. 17 in the main text], respectively. (**B**) Putative secondary structure for *BNAT1*, estimated by the *in silico* analysis based on minimum free energy prediction in RNAfold web server operated by University of Vienna [http://rna.tbi.univie.ac.at//cgi-bin/RNAWebSuite/RNAfold.cgi].

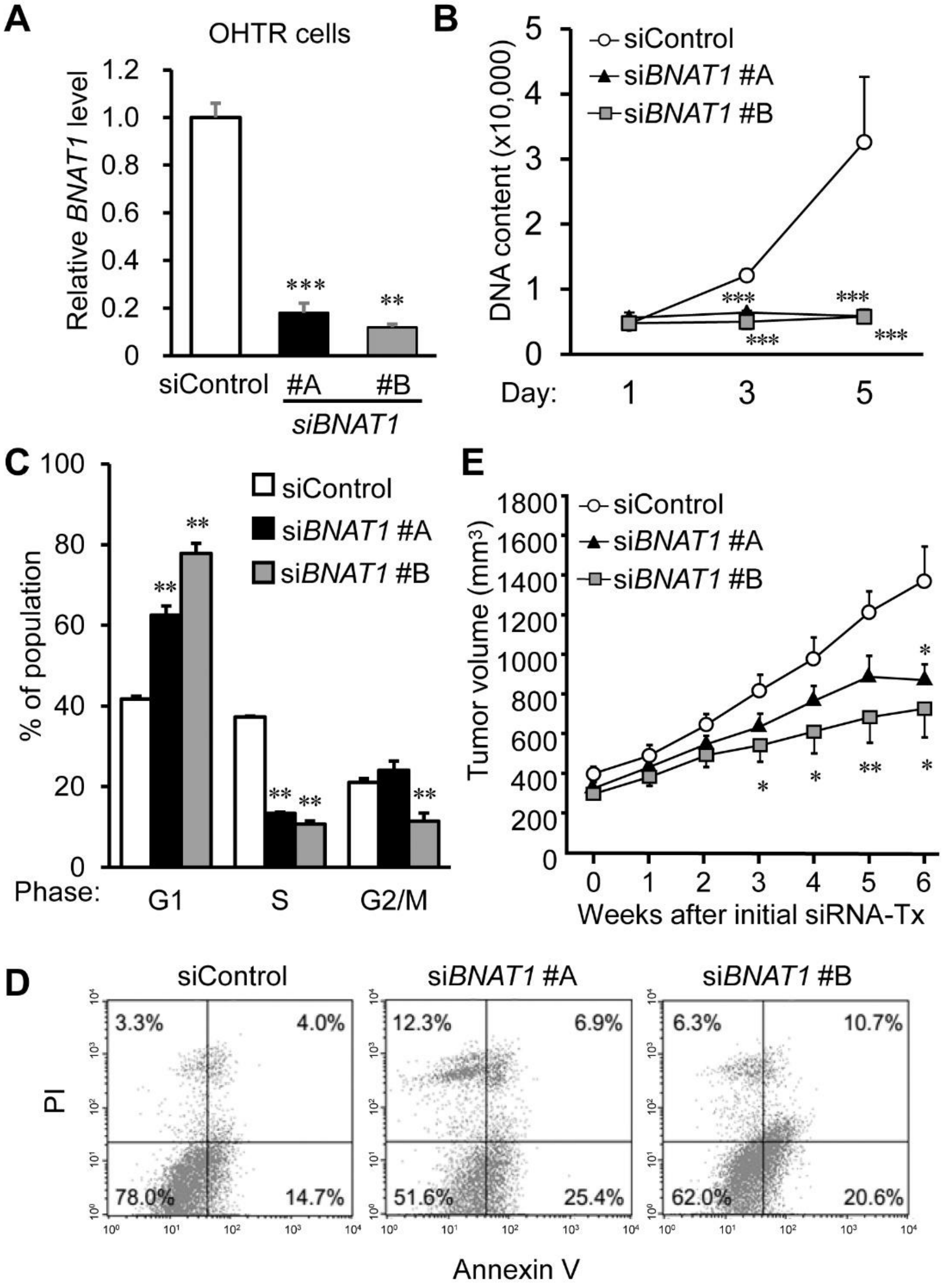

Figure S2. *BNAT1* silencing represses the viability and cell cycle progression, whereas promotes apoptosis of hormone-refractory breast cancer OHTR cells. (**A**) Knockdown efficiency of *BNAT1* siRNAs (si*BNAT1*s) #A and #B compared with control siRNA (siControl) in hormone-refractory OHTR cells analyzed by qRT-PCR (n = 3). (**B**) The viability of OHTR cells treated with indicated siRNAs, analyzed by DNA assay. Values are presented as means ± SEM *versus* levels for siControl in each cell type (n = 5). (**C**) Cell cycle distribution of siRNA-treated OHTR cells stained with propidium iodide (PI) analyzed by flow cytometry. Percentages of cell populations in G1, S, and G2/M phases are shown (n = 3). (**D**) Percentages of annexin V-positive populations in OHTR cells treated with the indicated siRNAs, analyzed by flow cytometry (n = 3). (**E**) *BNAT1*-specific siRNAs inhibit *in vivo* tumor growth of hormone-refractory breast cancer cells. OHTR cells were injected into flanks of nude mice and the indicated siRNAs were injected twice weekly into the developed xenograft tumors (n = 7 for siControl and si*BNAT1* #B groups and n = 9 for si*BNAT1* #A group). Tumor volumes are presented as means ± SEM. *, $p < 0.05$; **, $p < 0.01$; ***, $p < 0.001$.

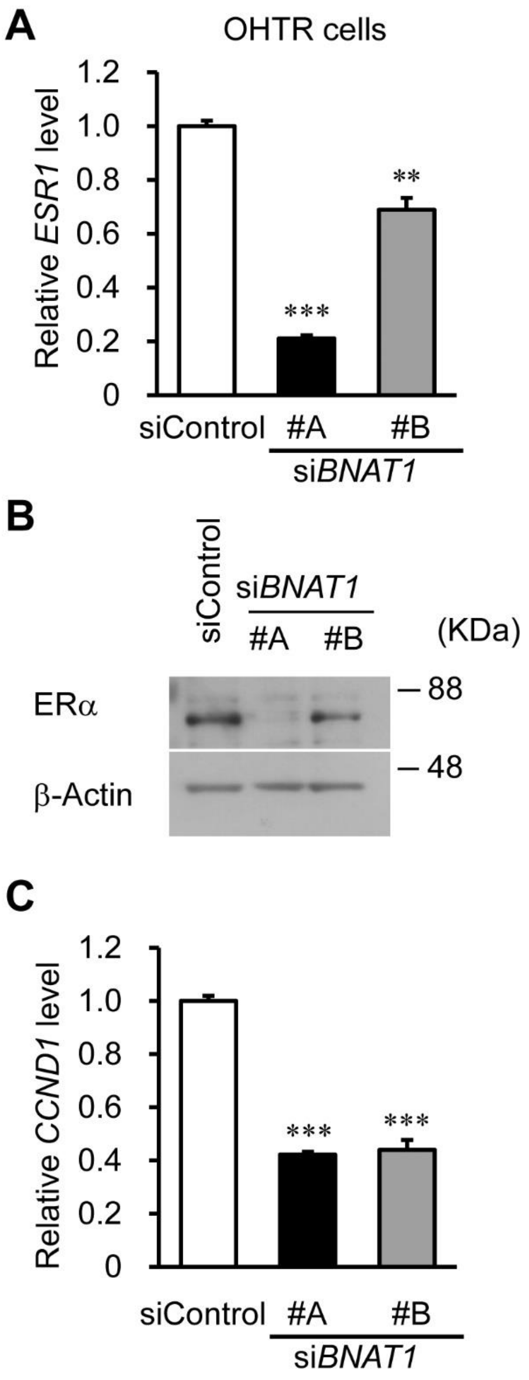

Figure S3. Effects of *BNAT1* silencing on the expression of ERα and its target gene *CCND1* in hormone-refractive OHTR cells. (**A**) Downregulation of *ESR1* expression in OHTR cells treated with si*BNAT1*s. Relative RNA levels are presented as mean fold changes ± SEM *versus* levels for siControl (n = 3). (**B**) Immunoblotting of ERα protein in OHTR cells treated with si*BNAT1*s. (**C**) Effect of *BNAT1* silencing on *CCND1* level in OHTR cells. Relative RNA levels are presented as mean fold changes ± SEM *versus* levels for siControl (n = 3). **, $p < 0.01$; ***, $p < 0.001$.

Table S1. Estrogen-inducible active enhancer clusters identified from a subgroup of ERα/acetylated histone H3K27 co-bound enhancers in MCF7 cells previously published in the literature [refs.6-8 in the main text]. These enhancers were shown to have following features: the association with estradiol (E_2)-upregulated enhancer RNAs, high intensity of ERα binding, and close proximity to estrogen target genes. Among the original 1,248 E_2-upregulated active enhancers, we focused on 66 clusters including at least 3 active enhancers per a region, with a distance between 2 adjacent enhancers ≤25 kb.

Chr	Start	End	Chr	Start	End
chr1	16,934,849	16,941,132	chr12	6,372,442	6,387,999
chr1	42,325,987	42,334,136	chr12	53,360,226	53,366,102
chr1	202,075,557	202,101,968	chr12	69,186,965	69,198,324
chr1	207,046,219	207,083,229	chr14	75,721,796	75,742,242
chr2	11,638,754	11,672,797	chr14	93,478,031	93,512,598
chr2	100,226,367	100,253,406	chr14	105,498,842	105,516,984
chr2	216,844,387	216,847,609	chr14	106,134,752	106,146,352
chr3	14,712,421	14,718,803	chr15	63,672,125	63,688,163
chr3	107,705,503	107,725,311	chr15	71,371,485	71,397,318
chr3	150,440,610	150,481,106	chr15	89,663,147	89,682,829
chr3	170,038,368	170,061,984	chr16	1,337,730	1,347,194
chr3	193,546,271	193,586,766	chr16	33,955,682	33,964,137
chr3	193,855,480	193,877,028	chr16	54,458,081	54,459,808
chr4	77,483,480	77,491,497	chr16	72,947,315	72,970,174
chr5	55,749,953	55,776,290	chr16	85,316,047	85,345,752
chr5	66,510,961	66,532,904	chr16	85,487,467	85,525,878
chr6	11,047,050	11,067,846	chr16	88,990,287	88,995,126
chr6	39,234,599	39,243,507	chr17	26,852,406	26,864,131
chr6	125,519,136	125,526,509	chr17	38,705,600	38,711,997
chr8	37,448,210	37,456,380	chr17	49,021,801	49,027,624
chr8	67,424,590	67,435,001	chr17	55,418,023	55,435,025
chr8	102,475,038	102,515,536	chr17	72,741,009	72,766,198
chr8	128,801,087	128,813,288	chr17	77,002,040	77,013,934
chr8	128,872,114	128,883,110	chr18	46,501,024	46,523,573
chr8	129,154,205	129,165,535	chr18	60,893,036	60,927,046
chr9	33,221,315	33,234,439	chr19	35,806,914	35,810,241
chr9	97,754,320	97,766,484	chr20	35,893,182	35,908,232
chr10	134,376	173,195	chr20	52,720,726	52,743,946
chr10	127,313,412	127,323,049	chr21	41,690,518	41,712,592
chr11	1,849,851	1,850,069	chr21	43,795,659	43,813,118
chr11	69,330,109	69,330,327	chr21	46,806,706	46,841,893
chr11	72,903,470	72,911,612	chr22	29,209,791	29,220,052
chr11	77,856,839	77,867,681	chr22	37,574,422	37,594,098
